# The link between ferroptosis and airway inflammatory diseases: A novel target for treatment

**DOI:** 10.3389/fmolb.2022.985571

**Published:** 2022-08-17

**Authors:** Zhiwei Lin, Xiaojing Yang, Lili Guan, Lijie Qin, Jiabin Ding, Luqian Zhou

**Affiliations:** State Key Laboratory of Respiratory Disease, National Clinical Research Center for Respiratory Disease, Guangzhou Institute of Respiratory Health, First Affiliated Hospital of Guangzhou Medical University, Guangzhou, China

**Keywords:** ferroptosis, metabolic networks and pathways, lung diseases, cell death, COVID-19

## Abstract

Ferroptosis is an iron-dependent mode of cell death characterized by intracellular lipid peroxide accumulation and a redox reaction imbalance. Compared with other modes of cell death, ferroptosis has specific biological and morphological features. The iron-dependent lipid peroxidation accumulation is manifested explicitly in the abnormal metabolism of intracellular lipid oxides catalyzed by excessive iron ions with the production of many reactive oxygen species and over-oxidization of polyunsaturated fatty acids. Recent studies have shown that various diseases, which include intestinal diseases and cancer, are associated with ferroptosis, but few studies are related to airway inflammatory diseases. This review provides a comprehensive analysis of the primary damage mechanisms of ferroptosis and summarizes the relationship between ferroptosis and airway inflammatory diseases. In addition to common acute and chronic airway inflammatory diseases, we also focus on the progress of research on COVID-19 in relation to ferroptosis. New therapeutic approaches and current issues to be addressed in the treatment of inflammatory airway diseases using ferroptosis are further proposed.

## 1 Introduction

Ferroptosis is a novel regulated cell death pathway discovered by Dixon et al. (Dixon et al., 2012) in 2012. Compared with other modes of cell death, such as apoptosis, necrosis, and autophagy, ferroptosis is mainly characterized by the accumulation of iron-dependent lipid peroxidation with specific features of abnormal metabolism of intracellular lipid oxides catalyzed by excess iron ions, reactive oxygen species (ROS) production, and regulatory cell death mediated by excessive oxidation of polyunsaturated fatty acids (PUFAs). The development and progression of many human diseases are related to ferroptosis (Fuchs and Steller, 2011), such as intestinal diseases (Xu et al., 2021) and tumor cell death (Xu et al., 2019).

Today there is growing evidence that ferroptosis is closely linked to inflammatory diseases (Mao et al., 2020). Airway inflammation is the body’s natural response to injury and it serves to remove harmful stimuli such as pathogens, irritants and damaged cells, and to initiate the healing process (Ahmed, 2011). The main airway inflammatory diseases are acute or chronic airway inflammation and infectious inflammatory diseases (Aghasafari et al., 2019). The main acute airway inflammatory diseases are acute lung injury and acute respiratory distress syndrome, while the main chronic airway inflammatory diseases are chronic obstructive pulmonary disease (COPD) and asthma (Racanelli et al., 2018; Aghasafari et al., 2019). In addition to these, there are also infectious airway inflammatory diseases of the airways such as *Pseudomonas aeruginosa* infection, *Mycobacterium tuberculosis* infection, acute bronchiolitis and the current epidemic of COVID-19 (Dorhoi and Kaufmann, 2014; Jartti et al., 2019; Berlin et al., 2020; Garcia-Clemente et al., 2020). To date, convincing evidence suggests that ferroptosis plays an important role in inflammation and a number of antioxidants that act as inhibitors of ferroptosis have been shown to exert anti-inflammatory effects in experimental models of certain diseases (Sun et al., 2020). However, the underlying mechanisms of ferroptosis in airway inflammatory diseases have not been fully elucidated and summarized. However, the underlying mechanisms have not been elucidated fully. This review systematically summarizes the latest progress in ferroptosis research and discusses the research progress of the relationship between ferroptosis and airway inflammatory diseases, which suggests new therapeutic approaches for airway inflammatory diseases.

## 2 Overview of ferroptosis

### 2.1 Iron balance and ferroptosis

Iron is a minor element required for the proper functioning of many physiological functions and is involved in the synthesis of many substances in the human body, such as hemoglobin, which transports oxygen from the lungs to other organs. Additionally, iron is a component of many enzymes and immune system compounds. The body obtains the most required iron from food and maintains the balance of iron metabolism. Iron in food exists mainly as trivalent iron ions that are reduced to ferrous ions by gastric acid, then form complexes with certain sugars, amino acids, and vitamin C in the gastrointestinal tract, which are absorbed in the duodenum and jejunum. In the average human body, iron is in a stable balance. Iron obtained from food can not only supplement the amount needed for human growth and development, but also make up for the amount lost in normal iron metabolism (Anderson et al., 2009; Lizarraga et al., 2009; Elsenhans et al., 2011).

Previous studies have shown that various mechanisms, such as necrosis, apoptosis, and autophagy, jointly regulate and control the death of all mammalian cells. However, in 2003, Sonam, DeHart, et al. (Dolma et al., 2003; DeHart et al., 2018) indicated that the anti-tumor drug erastin induces tumor cells to die uniquely. Further found under light microscopy, this novel manner of cell death exhibited a classical necrotic morphology accompanied by reduced or absent intracellular mitochondrial cristae and ruptured outer membranes with smaller intracellular mitochondria, an increased density of bilayer membranes. Furthermore, none of the characteristic changes expected of cell death, such as cytoplasmic swelling, rupture, chromatin condensation, and margination, were present. Nicholas Yagoda (Yagoda et al., 2007) in 2007 and Yang et al. (Yang and Stockwell, 2008) in 2008 found that the novel cell death described above was inhibited by iron chelators and accompanied by increased levels of reactive oxygen species. In 2012, Dixon et al. (Dixon et al., 2012) officially named it “ferroptosis” which is a non-apoptotic, iron-dependent mode of cell death mainly characterized by accumulation of intracellular reactive oxygen species.

The primary mechanism of ferroptosis is dependent on iron, which causes lipid peroxidation of cell membranes by disrupting the antioxidant system that consists of glutathione (GSH) and glutathione peroxidase (GPx) 4. The normal structural integrity of cells is disrupted and cell death occurs as the result. It is unknown whether ferroptosis is involved in the development of various diseases. However, some research suggested that ferroptosis is a physiological process that occurs widely in mammals rather than a pathological or organ-specific process (Linkermann et al., 2014; Zille et al., 2017). Unlike other forms of cell death, ferroptosis shares some features with several types of regulated cell death (RCD).

### 2.2 Mechanism and regulation of ferroptosis damage

#### 2.2.1 System Xc- pathway

Ferroptosis is a regulated cell death due to excess iron aggregation, which leads to an explosion of intracellular lipid-based reactive oxygen species. Simultaneously, it disrupts the antioxidant mechanism in the organism and large amounts of reactive oxygen species cause further lipid peroxidation and eventually cell death (Le et al., 2021). Cystine/glutamate transporter (system Xc-), a transmembrane amino acid transporter on the cell surface, consists of a heterodimer of two amino acid chains: the SLC3A2 heavy chain and SLC7A11 light chain, which transports cystine into the cell. Glutathione is an essential cofactor for glutathione peroxidases (GPxs) and glutathione peroxidases catalyze degradation of hydrogen peroxide (H_2_O_2_) with peroxides and inhibit lipid-based reactive oxygen species production (Bridges et al., 2012). Therefore, inhibiting cystine-glutamate transporter receptor and affecting glutathione production reduce glutathione peroxidases activity, which decreases the cellular antioxidant capacity as well as leading to lipid-based reactive oxygen species accumulation and causes cellular ferroptosis. Moreover, SLC7A11 directly promotes translational expression of glutathione peroxidase 4 protein through cystine and demonstrated that mTORC1 positively regulates the translational expression of glutathione peroxidase 4 protein by sensing cysteine levels through the small G protein Rag and relies on phosphorylation to inhibit 4EBP (Zhang et al., 2021).

#### 2.2.2 GPx4 pathway

GPx4 is a member of the glutathione peroxidase family and plays a crucial role in maintaining intracellular redox homeostasis. GPx4 catabolizes certain lipid peroxides and small molecule peroxides, which inhibits lipid peroxidation (Imai et al., 2017). Cells with reduced GPx4 expression have increased sensitivity to ferroptosis (Yang et al., 2014). Thus, inhibition of GPx4 expression causes cellular ferroptosis. GPx4 contains eight nucleophilic amino acids. Selenocysteine is an amino acid in the active center of GPx4. Inserting selenocysteine into GPx4 requires a special transporter, selenocysteine tRNA (Yang et al., 2016; Kagan et al., 2017). Maturation of selenocysteine tRNA requires isopentenyl transferase to transfer the isopentenyl group from isopentenyl pyrophosphate (IPP) to the selenocysteine tRNA precursor. isopentenyl pyrophosphate is the product of the mevalonate pathway, which acts on GPx4 by regulating the maturation of selenocysteine tRNA (Warner et al., 2000). Therefore, inactivation or deletion of GPx4 leads to the accumulation of lipid peroxides and causes ferroptosis in cells.

#### 2.2.3 p53 pathway

Jiang et al. first revealed that p53 can promote ferroptosis in cells by the mechanism that p53 can transcriptionally repress SLC7A11 expression (Jiang et al., 2015). Further studies have shown that acetylation of p53 K101 is of importance for p53 to inhibit SLC7A11 (Jiang et al., 2015). p53^3KR^ is an acetylation-deficient mutant form of p53 protein that does not induce cell cycle arrest, senescence and apoptotic processes, but fully retains the ability to regulate SLC7A11 expression and can contribute to cellular ferroptosis processes in a ROS-induced stress state (Jiang et al., 2015). The p53-SLC7A11 axis can also promote ferroptosis in a glutathione-independent manner. In one study, p53 was found to induce the expression of SAT1, which promotes the function of ALOX15, a member of the ALOX family, to enhance cellular ferroptosis (Ou et al., 2016). The ALOX12 is a key regulator of p53-dependent ferroptosis, and SLC7A11 can bind ALOX12 directly to limit its function, thus releasing ALOX12 when p53 inhibits SLC7A11 (Chu et al., 2019). Free ALOX12 can oxidize the polyunsaturated fatty acid chains of cell membrane phospholipids, leading to ferroptosis (Chu et al., 2019). In addition, the target gene of p53, GLS2, catalyzes the process of glutaminolysis (Suzuki et al., 2010), and glutaminolysis can promote the occurrence of ferroptosis (Yang et al., 2014). Meanwhile, p53 is able to regulate PHGDH to inhibit serine synthesis and may be able to influence glutathione synthesis to promote ferroptosis (Ou et al., 2015). In addition to the above, there are many other factors that can regulate p53 and further promote or inhibit ferroptosis. In conclusion, p53 is an important ferroptosis regulator with a broad and complex role (Liu and Gu, 2021; Liu and Gu, 2022).

#### 2.2.4 GCH1-BH4 pathway

Tetrahydrobiopterin (BH4) is a redox-active cofactor involved in the production of nitric oxide, neurotransmitters, and aromatic amino acids (Cronin et al., 2018). GTP cyclohydrolase-1- (GCH1-) 6-pyruvoyltetrahydropterin synthase- (PTS-) sepiapterin reductase (SPR) pathway catalyzes the conversion of GTP to BH4, and GCH1 is the rate-limiting enzyme in BH4 synthesis (Latremoliere and Costigan, 2011). BH4 exhibits antioxidant properties *in vitro* (Kim and Han, 2020). Kraft et al. identified GCH1 and its metabolic derivative BH4 as potent endogenous ferroptosis inhibitors that act independently of GPX4 pathway through a CRISPR activation screen (Kraft et al., 2020). GCH1 overexpression is resistant to RSL3-induced ferroptosis and gene ablation-induced ferroptosis by GPX4, but does not protect cells from apoptosis inducers, and and had only a weak effect on necrotic death, suggesting that GCH1 selectively resists cells from undergoing ferroptosis (Kraft et al., 2020). Soula et al. (2020) demonstrated that deletion of GCH1 or SPR, as well as inhibition of SPR with QM385, sensitized cells to RSL3 but not to Erastin treatment in Jurkat cells. Treatment of cells with BH2 or BH4 together with ferroptosis inducers saved cells from ferroptosis (Kraft et al., 2020; Soula et al., 2020). Although BH4 can act as a cofactor for several biosynthetic enzymes, both groups found this function of BH4 to be independent of its protective effect against ferroptosis (Kraft et al., 2020; Soula et al., 2020). The results of both studies suggest that the GCH1-BH4 pathway acts as an endogenous antioxidant pathway to inhibit ferroptosis by acting independently of the GPX4/glutathione mechanism. However, the role of BH4 in ferroptosis still needs to be further confirmed under pathological conditions.

#### 2.2.5 Methionine pathway

Under oxidative stress conditions, the sulfur transfer pathway transfers sulfur atoms from methionine to serine to produce cysteine. Cysteine is then used as a substrate to bind glutamate and glycine to synthesize glutathione, thereby synthesizing glutathione peroxidases to maintain intracellular redox homeostasis and prevent oxidative damage. Therefore, this pathway inhibits the occurrence of ferroptosis (McBean, 2012).

#### 2.2.6 Other pathways

A mixture of hydrogen peroxide and divalent iron ions is strongly oxidizing and its main oxidizing component is hydroxyl radicals (OH·) (Winterbourn, 1995). When excessive iron ions were enriched in an organism, the circulating non-transferrin-bound iron (NTBI) content increased, which reacted with hydrogen peroxide to induce the Fenton reaction, accumulate reactive oxygen species and a large amount of hydroxyl radicals, and oxidize polyunsaturated fatty acids on the cell membrane to lipid hydroperoxide (LPO) (Pignatello et al., 2006). Lipid hydroperoxide forms toxic lipid radicals, such as alkoxy radicals, which damage the structural stability of cell membranes and attack intracellular DNA and proteins to cause ferroptosis in cells. Additionally, these radicals transfer protons from neighboring polyunsaturated fatty acids, which initiates a new round of lipid oxidation reactions and further transmits oxidative damage (Shah et al., 2018).

Voltage-dependent anion channels (VDACs) are ion channels located on the outer mitochondrial membrane, which mediate and control the exchange of molecules and ions between mitochondria and the cytoplasm (Mazure, 2017; Becker and Wagner, 2018). VDACs permeability can be altered by drugs, which disrupts mitochondrial metabolism, generates large amounts of reactive oxygen species, and leads to cellular ferroptosis (Maldonado, 2017).

Ferroptosis is inhibited by FSP1 overexpression, but when CoQ10 is simultaneously deficient, the ability of cells to inhibit ferroptosis disappears. FSP1 prevents lipid oxidation by reducing CoQ10 (Doll et al., 2019). Another study (Bersuker et al., 2019) also showed that FSP1 inhibits ferroptosis through reducing CoQ10 by mutating the conserved glutamate bound by cofactors in FSP1, which was independent of the classical GPX4 signaling pathway. The most recent study (Mao et al., 2021) found that DHODH is an enzyme located on the outer surface of the mitochondrial intima, which inhibits ferroptosis by reducing CoQ to COQH_2_, and further proposed a defense mechanism of DHODH-mediated mitochondrial ferroptosis.

**FIGURE 1:**
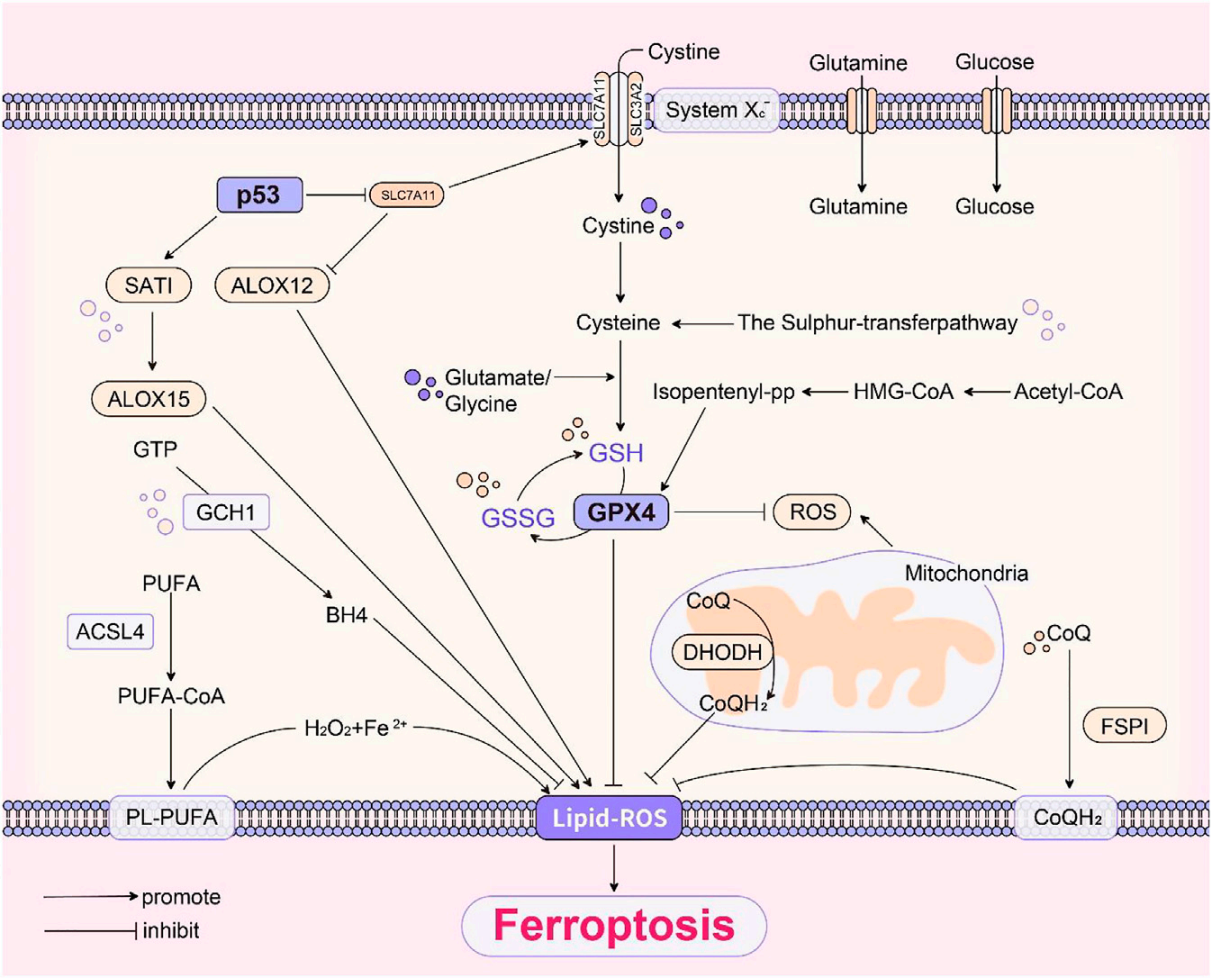
(The basic pathways of ferroptosis).

Based on all the above mechanisms, Figure 1 summarised the basic pathways of ferroptosis.

## 3 Ferroptosis and acute airway inflammatory diseases

Acute lung injury (ALI) and the more severe acute respiratory distress syndrome are pulmonary manifestations of an imperative systemic inflammatory process with clinical presentations of pulmonary infiltration, diffuse alveolar injury, hypoxemia, and pulmonary edema formation (Butt et al., 2016).

In an oleic acid (OA)-induced acute lung injury mouse model, the lung histopathological score, lung dry weight ratio, and protein content were elevated in the bronchoalveolar lavage fluid (BALF) of the oleic acid group compared with the control group, and mitochondrial contraction and mitochondrial membrane rupture were seen in lung cells. Moreover, there was iron overload, glutathione depletion, accumulation of malondialdehyde (MDA), downregulation of protein expression levels of GPx4 and ferritin in lung tissue, and 7-fold higher expression levels of PTGS2 mRNA in the oleic acid group than in the control group. These results suggest that ferroptosis plays an essential role in the pathogenesis of acute lung injury (Zhou et al., 2019). *In vivo* and *in vitro* that lipopolysaccharide (LPS) reduces SLC7A11 and GPx4 with elevated levels of malondialdehyde, 4-hydroxynonenal (4-HNE), and total iron in human bronchial epithelial cell line BEAS-2B, thereby causing ferroptosis in these cells. Ferrostatin-1 effectively alleviates the decrease in BEAS-2B cell activity caused by lipopolysaccharide and reduces lung inflammation by inhibiting ferroptosis (Liu et al., 2020). Therefore, ferroptosis may be a new therapeutic target for acute lung injury and more suitable ferroptosis inhibitors can be investigated to alleviate the progression of inflammation in acute lung injury.

Characteristic mitochondrial morphological changes caused by ferroptosis in type II alveolar epithelial cells of an intestinal ischemia/reperfusion-induced acute lung injury (IIR-ALI) model in Nrf2 gene knockout C57BL/6 mice. After treating the mice with Fe and ferrostatin-1, the former significantly aggravated pathological damage, pulmonary edema, lipid peroxidation, and promoted cell death. The latter had the opposite effects (Dong et al., 2020). In the oxygen-glucose deprivation and reoxygenation (OGD/R) model, MLE12 and BEAS-2B cells increase expression levels of Nrf2, SLC7A11, and heme oxygenase-1 (HO-1) proteins. After treating mice with Fe and ferrostatin-1, The results also showed that the former promoted the occurrence of ferroptosis, while the latter inhibited it (Lee et al., 2014; Dong et al., 2020). Knockdown of Nrf2 significantly inhibits the expression of SLC7A11 and Heme oxygenase-1 (Dong et al., 2020). Thus, Nrf2 inhibits ferroptosis in alveolar epithelial cells by regulating SLC7A11 and heme oxygenase-1, which provides a potential therapeutic strategy for intestinal ischemia/reperfusion-induced acute lung injury. Nrf2 mitigates the progression of intestinal ischemia/reperfusion-induced acute lung injury by mediating STAT3 activation in an oxygen-glucose deprivation and reoxygenation model established with MLE12 cells (Qiang et al., 2020). Lung ischemia-reperfusion (Lung-IR) increases tissue iron content and lipid peroxidation accumulation during reperfusion and increases the expression of essential proteins (GPx4 and ACSL4). In animal and cellular models, pretreatment with liproxstatin-1 inhibits ferroptosis and ameliorates lung damage caused by lung ischemia-reperfusion. Additionally, preischemic administration of the ACSL4 inhibitor rosiglitazone attenuates the occurrence of ferroptosis in lung tissue with ischemia-reperfusion, which is consistent with the protective effect of ACSL4 knockdown on lung epithelial cells that have experienced hypoxia/reoxygenation (Xu et al., 2020). Thus, ACSL4 is associated with ferroptosis in lung tissue induced by ischemia-reperfusion.

Reactive oxygen species levels in the lung and serum levels of inflammatory cytokines (TNF-α, IL-6, IL-10, and TGF-β1) were significantly reduced in mice treated with ferroptosis inhibitors after acute radiation-induced lung injury (RILI) (Li et al., 2019). This suggests that radiation-induced reactive oxygen species is the initial trigger of ferroptosis in acute radiation-induced lung injury and that ferroptosis may also be important in affecting inflammatory cytokine levels in acute radiation-induced lung injury. Sevoflurane protected mice from LPS-induced lung injury, which included reductions in lung histological damage, pulmonary edema, and pulmonary vascular permeability, and inflammatory factors in bronchoalveolar lavage fluid as well as improving the survival of acute lung injury mice, which are consistent with the action of the ferroptosis inhibitor ferrostatin-1 (Liu et al., 2021). Further experiments revealed that Sevoflurane alleviates LPS-induced acute lung injury by upregulating heme oxygenase-1 expression to inhibit ferroptosis.

## 4 Ferroptosis and chronic airway inflammatory diseases

Studies of ferroptosis in chronic airway inflammatory diseases have focused on chronic obstructive pulmonary disease and bronchial asthma. However, systematic and comprehensive studies have not been conducted in detail.

### 4.1 Ferroptosis and chronic obstructive pulmonary disease

The pathogenesis of the chronic obstructive pulmonary disease (COPD) is associated with disturbances in iron homeostasis, which lead to excessive oxidative stress (Yoshida et al., 2019). Further studies showed that ferroptosis is related to chronic obstructive pulmonary disease pathogenesis. *In vivo* and *ex vivo* models (Gao et al., 2016; Hou et al., 2016) have shown that cigarette smoke (CS) exposure enhances unstable iron accumulation and lipid peroxidation in human bronchial epithelial cells (HBECs). Moreover, the cells initiate NCOA4-mediated ferritinophagy in response to CS-induced ferritin degradation, which increases free iron content and promotes ferroptosis in cells. In a cigarette smoke exposure model, chronic obstructive pulmonary disease mice with GPx4 knockout showed an increase in the lipid peroxide level. Additionally, they had an increase in small airway thickness compared with normal mice with corresponding symptoms detected in the lung tissue of smokers. In addition to causing cellular ferroptosis, damage-associated molecular patterns (DAMPs) and proinflammatory cytokine release from lung epithelial cells were also detected in the above mouse model, which exacerbates the degree of peripheral inflammation. This study further suggested that the decrease in GPx4 and its substrate glutathione may be related to the inadequate antioxidant stress response of human bronchial epithelial cells to cigarette smoke, which leads to ferroptosis. NCOA4 expression levels are significantly higher in lung homogenates from chronic obstructive pulmonary disease patients than non-smokers and non-chronic obstructive pulmonary disease smokers. Immunohistochemistry of lung tissue also showed enhanced expression of NCOA4 in bronchial epithelial cells of chronic obstructive pulmonary disease patients. Taken together, these findings support the role of CS-induced ferroptosis in the pathogenesis of chronic obstructive pulmonary disease. Particulate matter 2.5 (PM2.5) also causes chronic airway inflammation because of ferroptosis in endothelial cells, which is similar to what occurs after cigarette smoke exposure (Wang and Tang, 2019). Thus, new therapeutic ideas can be explored to alleviate chronic obstructive pulmonary disease symptoms by regulating unstable iron content and the lipid peroxidation response in human bronchial epithelial cells.

In healthy airways, alveolar macrophages (AMs) are relatively quiescent. However, in chronic obstructive pulmonary disease, various etiologies induce increased activation and numbers of alveolar macrophages that produce proinflammatory mediators to induce neutrophils, monocytes, and T cells to enter the lungs and exacerbate lung tissue injury and inflammation (Christenson, 2017). Iron overload-induced macrophages undergo ferroptosis, iron citrate induces ferroptosis in bone marrow-derived macrophages, and SLC7A11 gene deletion promotes iron overload-induced ferroptosis in macrophages (Wang et al., 2017). Additionally, macrophages recognize oxidized phospholipids on the surface of iron-dead cells through the membrane receptor toll-like receptor 2, which mediates phagocytic clearance of ferroptotic cells (Luo et al., 2021). Therefore, ferroptosis may be induced in cells and thus reduce airway inflammation, which requires further investigation.

### 4.2 Ferroptosis and bronchial asthma

Reactive oxygen species production in peripheral blood eosinophils is significantly higher in asthmatic patients than in healthy subjects and that reactive oxygen species production is further increased dramatically after excitation (Frossi et al., 2008). On the other hand, inflammation in asthma may also be associated with damage-associated molecular patterns. Tissues with ferroptosis suggest significant activation of macrophages and release of pro-inflammatory substances, which triggers a series of inflammatory responses. Additionally, there are inflammatory mediators produced by the metabolism of peroxides and arachidonic acid in ferroptosis tissues (Shah et al., 2018). As a critical lipid peroxidase in arachidonic acid metabolism, lipoxygenase is not only involved in the inflammatory and immune responses of the body but also catalyzes the oxidation of polyunsaturated fatty acids to lipid peroxides, which forms toxic lipid free radicals that damage the structural stability of cell membranes and attack intracellular DNA and proteins, thereby causing ferroptosis in cells. Furthermore, these free radicals transfer protons from neighboring PUFAs, initiate a new round of lipid oxidation reactions and impart further oxidative damage.

Establishing a mouse ovalbumin asthma model and performed experiments using ferroptosis inducers (FINs) *in vivo* and *in vitro* to detect the survival of eosinophils and the level of inflammation in lungs, which found that ferroptosis inducers mediate eosinophil ferroptosis through a non-classical pathway (cytoplasmic reactive oxygen species accumulation), thereby effectively alleviating asthma with eosinophilic airway inflammation. Furthermore, antioxidants glutathione and N-acetylcysteine significantly attenuated FIN-induced cell death and reduced eosinophilic airway inflammation in mice. There was also a significant synergistic effect between ferroptosis of eosinophils induced by ferroptosis inducers and apoptosis of eosinophils induced by glucocorticoids (Wu et al., 2020). Thus, their combined use can further induce the death of eosinophils, reduce the application of hormones, and further protect asthmatic airways. Thus, eosinophil apoptosis may provide a new target for treating eosinophilic airway inflammation and a new therapeutic option for clinically hormone-tolerant or refractory asthma patients, which requires further investigation.

PEBP1 is essential for the dynamic balance between the ferroptosis program and cellular autophagy in asthmatic airway epithelial cells and activation of autophagy protects cells from ferroptosis and mitochondrial DNA release. Similar findings were observed in type 2 high phenotype asthma epithelial cells. The finding that the 15-lipoxygenase-1-PEBP1 (15LO1-PEBP1) complex and its phospholipid hydroperoxide are accompanied by ferroptosis and autophagy activation revealed a pathobiological pathway associated with asthma (Zhao et al., 2020). Therefore, ferroptosis is closely related to the mechanism of bronchial asthma exacerbation, which provides new ideas to improve the prognosis of asthma.

## 5 Ferroptosis and infectious airway inflammatory diseases

### 5.1 Ferroptosis and *P*. *aeruginosa* infection

*P. aeruginosa* expresses lipoxygenase (pLoxA) that selectively oxidizes arachidonic acid-phosphatidyl ethanolamine (AA-PE) to produce 15-hydroperoxy-AA-PE (15-HO-AA-PE). The accumulation of 15-HOO-AA-PE among other molecules causes cellular ferroptosis as a ferroptosis inducer. Although *P. aeruginosa* itself has no AA-PE on its cell membrane, the synthesized lipoxygenase can target the transformation of arachidonic acid in the cell membrane of host cells, which is similar to the body’s mechanism and causes the onset of ferroptosis in host cells. In the clinic, high expression of lipoxygenase causes ferroptosis in bronchial epithelial cells with *P. aeruginosa* colonization. Conversely, mutant bacteria that lack lipoxygenase do not cause ferroptosis in human bronchial epithelial cells (Dar et al., 2018). Thus, isolation of *P. aeruginosa* in patients with chronic lower respiratory tract infections depends on the expression level and enzymatic activity of lipoxygenase. On the basis of these findings, the development of specific lipoxygenase inhibitors may be a novel therapy for chronic lower respiratory tract infections by *P. aeruginosa*.

*P. aeruginosa* degrades host GPx4 defenses by activating lysosomal chaperon-mediated autophagy (CMA). In response, the host stimulates the iNOS/NO^•^ driven anti-ferroptotic mechanism to prevent lipid peroxidation and protect GPx4/GSH-deficient cells. Macrophage production of NO^•^ as an inter-cellular mechanism was found to prevent PA-stimulated ferroptosis in epithelial cells at a distance using a co-culture model system. The inhibitory effect of NO^•^ on ferroptosis in epithelial cells was inhibited by inhibiting phospholipid peroxidation, especially the production of pro-ferroptotic 15-hydroperoxy- arachidonyl-PE (15-hPET-PE) signals. The pharmacological targeting of iNOS weakens its anti- ferroptosis function (Dar et al., 2021). Based on these studies, this could lead to new therapeutic strategies against ferroptosis induced by *P. aeruginosa*.

### 5.2 Ferroptosis and *M*. *tuberculosis* infection

*M. tuberculosis* (Mtb) infection increases the expression of heme oxygenase-1 that degrades heme to free iron (Andrade et al., 2013; Costa et al., 2016). Free iron levels are associated with an increased tuberculosis (TB) risk in patients. Additionally, elevated iron levels exacerbate lung inflammation and increase bacterial load in patients and animals with *M. tuberculosis* infection (Schaible et al., 2002; Boelaert et al., 2007). Mtb-induced macrophage death is associated with decreased glutathione and GPx4 levels and increased free iron, mitochondrial superoxide, and lipid peroxide, which are markers of ferroptosis. However, macrophage death after *M. tuberculosis* infection can be alleviated by ferrostatin-1 or iron chelators, which effectively reduces bacterial load and lung inflammation (Amaral et al., 2019). Therefore, appropriate treatments can be explored to inhibit host cell ferroptosis and thus alleviate tuberculosis. Of note during the COVID-19 epidemic is that human immune function can be temporarily suppressed due to the effects of SARS-CoV-2 and possibly immunosuppressive drugs, leading to reactivation of *M. tuberculosis* or infection causing active TB disease. We should be sensitive to the short-term increase in TB prevalence after the end of the COVID-19 pandemic. This requires not only the implementation of TB prevention and control, but also appropriate measures to enhance TB prevention, control and management (Yang and Lu, 2020).

### 5.3 Ferroptosis and acute bronchiolitis

Acute bronchiolitis is an acute infection of the lower respiratory tract resulting in obstruction or dyspnea, with major symptoms including cough, runny nose, paroxysmal wheezing, and in severe cases, hypoxemia, which affects infants and children worldwide (Dalziel et al., 2022). It is most often caused by human respiratory syncytial virus (RSV) and the lung epithelium and alveolar macrophages are the first cells to be infected during RSV infection (Blanco et al., 2002). It has been shown that RSV causes more than 30 million cases of lower respiratory tract infections in children under 5 years of age each year, with 32 million hospitalizations and 200,000 deaths worldwide each year (Bohmwald et al., 2016). Bronchiolitis is one of the main reasons of consultations in pediatric emergency department (Redant et al., 2021). Vahid Salimi et al. showed that RSV increases 12/15-LOX expression and mitochondrial iron content through experiments in mice (Salimi et al., 2017). Lipoxygenases (LOX) are a family of enzymes capable of binding oxygen to unsaturated fatty acids (Rossaint et al., 2012). The mechanism is that 12/15 LOX inhibits CISD1 (Wang M. P. et al., 2021), an iron-containing mitochondrial outer membrane protein that negatively regulates ferroptosis (Yuan et al., 2016). Knockdown of CISD1 by RNAi increases mitochondrial Fe^2+^ accumulation, which subsequently causes lipid peroxidation of mitochondrial membranes induced by Fe^2+^-mediated Fenton reaction, thus promoting ferroptosis (Yuan et al., 2016). Therefore, the 12/15-LOX/CISD1 pathway may be a candidate target for the prevention and treatment of RSV infection through ferroptosis, and other possible mechanisms for the interaction between the 12/15-LOX pathway and RSV pathogenesis need to be discussed, which requires further targeted studies.

### 5.4 Ferroptosis and COVID-19

COVID-19 is a global pandemic with SARS-CoV-2 as the causative agent, manifesting itself as a severe inflammatory response characterised by a cytokine storm that has now caused widespread loss of life (Yi et al., 2020). SARS-CoV-2 targets the respiratory system and is transmitted from potentially symptomatic or asymptomatic infected individuals through contact droplets and contaminants. During the incubation period, the virus triggers a slow response in the lungs. SARS-CoV-2 invades mainly alveolar epithelial cells, causing respiratory symptoms (Yi et al., 2020). Although many patients are asymptomatic or have mild symptoms such as fever, fatigue and dry cough, a few cases progress to more severe forms of the disease such as ARDS, mainly in older men with comorbidities (Borges do Nascimento et al., 2020). Despite the amount of time scientists around the world have spent studying COVID-19, there is still no way to control the widespread spread of the epidemic. Studies have shown that isolation of infected cases can be effective in controlling the spread of the disease (Wang et al., 2020).

**FIGURE 2:**
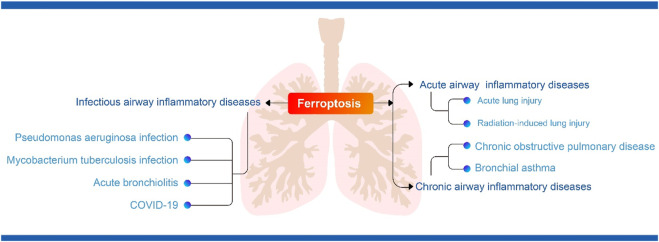
(The relationship between ferroptosis and airway inflammatory diseases).

There is now growing evidence that ferroptosis is involved in the pathogenesis of COVID-19 (Li et al., 2022). Ferroptosis has been detected in human heart (Jacobs et al., 2020) and hamster lung (Han et al., 2022) samples infected with SARS-CoV-2. A study showed that changes in markers of iron metabolism in blood samples, such as decreased serum iron and increased ferritin, were associated with severe COVID-19 (Zhou et al., 2020). Inflammatory manifestations following SARS-COV-2 infection can highly induce IL-6 secretion. Previous studies have demonstrated the effect of IL-6 on iron metabolism. On the one hand, IL-6 directly promotes transferrin uptake and ferritin expression (Kobune et al., 1994); on the other hand, IL-6 can induce the synthesis of ferroregulin (Nemeth et al., 2004), an inhibitor of iron transport proteins, which leads to cellular iron accumulation. Studies have shown that an increase in serum ferroregulin correlates with the severity of COVID-19 (Nai et al., 2021). In addition, scRNA-seq data from PBMC, T cells and B cells from COVID-19 patients showed that ferroptosis-related genes (including GPX4, FTH1, FTL and SAT1) were increased in the acute phase and decreased in the recovery phase (Huang et al., 2021). In addition, SARS-CoV-2 infection may lead to tissue depletion of selenium and transcriptional inactivation of GPX4, which synergistically disrupts GPX defences and induces the onset of ferroptosis (Wang Y. et al., 2021). In response to the ferroptosis manifestation of COVID-19, it has been suggested that drugs that enhance the GPX4-GSH axis, induce RTA and ACSL4 activity and ultimately lead to iron depletion in the unstable pool may be candidates for COVID-19 treatment (Fratta Pasini et al., 2021). In addition, the fact that Nrf2 directly or indirectly regulates antioxidant capacity and the HO-1-iron regulation-related axis suggests that NRF2 activators may be a new approach for the treatment of COVID-19 organ damage (Fratta Pasini et al., 2021). One study also identified iron chelators as useful adjunctive therapies in the treatment of COVID-19, such as deferasirox, desferrioxamine and deferiprone, as well as the naturally occurring iron chelator lactoferrin, which may be beneficial in combating COVID-19 disease progression (Perricone et al., 2020). Studies have shown that iron chelators not only chelate iron and reduce inflammation, but also prevent coronaviruses from binding to the receptors they use to enter host cells (Chang et al., 2020). To date, however, it has not been clinically established whether inhibition of ferroptosis is useful in the treatment of inflammatory storms and organ damage caused by SARS-CoV-2 infection, and more in-depth studies are needed to show the way forward. Figure 2 summarised the relationship between ferroptosis and airway inflammatory diseases.

## 6 Summary and future perspectives

The airway is a vital organ for oxygen entry into the body, and excessive inflammation can be life-threatening (Lumb, 2016). The delicate balance between inflammation and anti-inflammation is critical for airway homeostasis. Airway inflammation is usually caused by pathogens or exposure to toxins, pollutants, irritants and allergens (Lumb, 2016). Inflammatory target proteins that cause airway inflammation are diverse, such as matrix metalloproteinase-9 (MMP-9), intercellular adhesion molecule-1 (ICAM-1), vascular cell adhesion molecule-1 (VCAM-1), cyclooxygenase-2 (COX)-2) and cytoplasmic phospholipase A2 (cPLA2) (Lee and Yang, 2013), making airway inflammatory diseases diverse and complex. As a newly discovered programmed cell death pathway, ferroptosis has morphological and biochemical characteristics that are different from other cell death pathways such as apoptosis, necrosis and autophagy, and the specific mechanisms, related pathways and drug targets of ferroptosis for the development of airway inflammatory diseases still need to be further explored and studied. This review summarizes the recent progress of ferroptosis research and highlights that in addition to GPX4-dependent ferroptosis, the non-GPX4-dependent p53 regulatory network is also crucial for the regulation of ferroptosis. Furthermore, we comprehensively discuss the role of ferroptosis in various airway inflammatory diseases, including acute airway inflammatory diseases (acute lung injury), chronic airway inflammatory diseases (COPD, asthma) and infectious airway inflammatory diseases (*p. aeruginosa* infection, *M. tuberculosis* infection, acute bronchiolitis and COVID-19), to provide a new way of thinking for the treatment of airway inflammatory diseases.

Although various related studies are emerging, there are still many issues that need to be addressed before we can turn ferroptosis to relevant clinical applications. First, almost all current studies of airway inflammatory diseases only examine ferroptosis in cellular and animal models, and there is a lack of validated human evidence. Therefore, the design of clinical trials related to ferroptosis plays a decisive role. Second, the toxicological effects of inhibitors or inducers of ferroptosis on human organs are almost unknown. How can ferroptosis therapies be developed with higher efficacy and targeting, thus reducing overall systemic toxicity and improving safety? This remains to be addressed. Ferroptosis has two-sided effects in different diseases, with some diseases in which inhibition of ferroptosis can delay disease progression, such as COPD (Yoshida et al., 2019; Lin et al., 2022), while in tumors promoting ferroptosis can inhibit disease progression (Yan et al., 2021). We need to weigh this issue when using ferroptosis reagents in patients with multiple diseases at the same time in clinical practice. In the meantime, a large number of clinical studies are still needed to investigate the administration routes to be sure. Finally, there is crosstalk between ferroptosis and other cell death, such as cuproptosis, which has been widely debated recently (Tsvetkov et al., 2022). Further elucidation of the interrelationship between different cell death modalities is also necessary to explore the mechanisms involved and to develop therapeutic approaches.

In conclusion, ferroptosis, as a new mode of cell death, has considerable potential for research in airway inflammatory diseases, and with more in-depth exploration, it will certainly bring new strategies for the diagnosis and treatment of the disease. Given the complex pathology of various types of airway inflammatory diseases, although many preclinical studies have shown ferroptosis to be a promising drug target, the potential molecular signaling pathways and networks in various types of target cells in these diseases remain to be explored in depth.

## Glossary

ROS: Reactive oxygen species
PUFAs: Polyunsaturated fatty acids
GSH: Glutathione
GPx: Glutathione peroxidase
RCD: Regulated cell death
System Xc-: Cystine/glutamate transporter
GPxs: Glutathione peroxidases
H2O2: Hydrogen peroxide
IPP: Isopentenyl pyrophosphate
VDACs: Voltage-dependent anion channels
OH: Hydroxyl radicals
NTBI: Non-transferrin-bound iron
LPO: Lipid hydroperoxide
ALI: Acute lung injury
OA: Oleic acid
BALF: Bronchoalveolar lavage fluid
MDA: Malondialdehyde
LPS: Lipopolysaccharide
4-HNE: 4-hydroxynonenal
IIR-ALI: Intestinal ischemia/reperfusion-induced acute lung injury
OGD/R: Oxygen-glucose deprivation and reoxygenation
HO-1: Heme oxygenase-1
Lung-IR: Lung ischemia-reperfusion
RILI: Radiation-induced lung injury
pLoxA: Lipoxygenase
AA-PE: Arachidonic acid-phosphatidyl ethanolamine
15-HO-AA-PE: 15-hydroperoxy-AA-PE
CMA: Chaperon-mediated autophagy
15-hPET-PE: 15-hydroperoxy- arachidonyl-PE
Mtb: Mycobacterium tuberculosis
TB: Tuberculosis
COPD: Chronic obstructive pulmonary disease
CS: Cigarette smoke
HBECs: Human bronchial epithelial cells
DAMPs: Damage-associated molecular patterns
PM2.5: Particulate matter 2.5
AMs: Alveolar macrophages
FINs: Ferroptosis inducers
15LO1-PEBP1: 15-lipoxygenase-1-PEBP1
